# Cushing’s syndrome screening with the 1-mg dexamethasone suppression test in metabolically healthy and unhealthy obesity phenotypes

**DOI:** 10.1038/s41366-024-01598-0

**Published:** 2024-08-09

**Authors:** Sema Hepsen, Umran Gul, Hayri Bostan, Pinar Akhanli, Muhammed Erkam Sencar, Muhammed Kizilgul, Ilknur Ozturk Unsal, Erman Cakal

**Affiliations:** 1 Ankara Etlik City Hospital, Department of Endocrinology and Metabolism, Ankara, Turkey; 2 Canakkale Mehmet Akif Ersoy State Hospital, Department of Endocrinology and Metabolism, Canakkale, Turkey; 3 Erzurum Regional Training and Research Hospital, Department of Endocrinology and Metabolism, Erzurum, Turkey; 4 Medicana International Ankara Hospital, Department of Endocrinology and Metabolism Ankara, Ankara, Turkey

**Keywords:** Obesity, Metabolic syndrome

## Abstract

**Background:**

The ongoing debate regarding the need for screening Cushing’s syndrome (CS) in patients with obesity continues. The objectives of this study were to establish the prevalence of CS in the population with obesity and assess how metabolic health status influences cortisol levels following the 1 mg dexamethasone suppression test (DST).

**Methods:**

This retrospective study included 1008 patients with obesity who underwent screening with the 1 mg DST for CS. These patients were categorized into two groups as metabolically healthy obesity (MHO) and unhealthy obesity (MUO).

**Results:**

Out of the 1008 patients, 779 (77.3%) belonged to the MUO group. Within the entire study cohort, 12 (1.2%) patients exhibited a cortisol level of ≥ 1.8 after the 1 mg DST. Cortisol levels following the 1 mg DST were also significantly higher in the MUO group than in the MHO group (*p* = 0.001). Among these 12 patients, 11 were presenting a MUO phenotype. Hypercortisolism was definitively diagnosed in two patients, resulting in an overall prevalence of 0.2%. The 1 mg DST demonstrated a specificity of 99% and 100% sensitivity for screening for CS.

**Conclusions:**

While the 1 mg DST is a practical screening test for CS with high specificity in obesity, the number of CS cases detected remains relatively low. Therefore, it may be more reasonable and applicable to screen patients with MUO phenotype rather than all individuals with obesity.

## Introduction

Cushing’s syndrome (CS) is a rare endocrinological disorder that poses challenges in both diagnosis and management [1]. Frequently, the diagnosis of this condition is delayed until clinical signs and symptoms become more pronounced, often resulting in the emergence of significant comorbidities by that time [2]. The current guidelines recommend screening adult patients who display clinical conditions such as hypertension and osteoporosis not typically associated with their age, multiple clinical features consistent with CS, as well as patients with adrenal adenoma [3]. Obesity, as one of the most common clinical features of CS, can also be an easily misleading symptom due to its rapidly increasing prevalence in the general population. Therefore, clarifying the uncertainty regarding the need for regular screening for CS in patients with obesity becomes more important.

Previous data has shown that some individuals with obesity may remain free from obesity-related comorbidities regardless of their body mass index (BMI) [4, 5]. This has led to the establishment of a new phenotype known as metabolically healthy obesity (MHO), which defines individuals with obesity who do not exhibit any cardiovascular diseases, glucose metabolism impairments, hypertension, or dyslipidemia [6].

Several studies have investigated the indication for CS screening in different populations with obesity and have reported varying prevalences of CS [7, 8]. We aimed to evaluate the outcomes of CS screening using a 1 mg dexamethasone suppression test (DST) in patients with obesity and determine if there is a difference in these outcomes based on the patients’ metabolic health status.

## Material and methods

### Study design and subjects

**Fig. 1:**
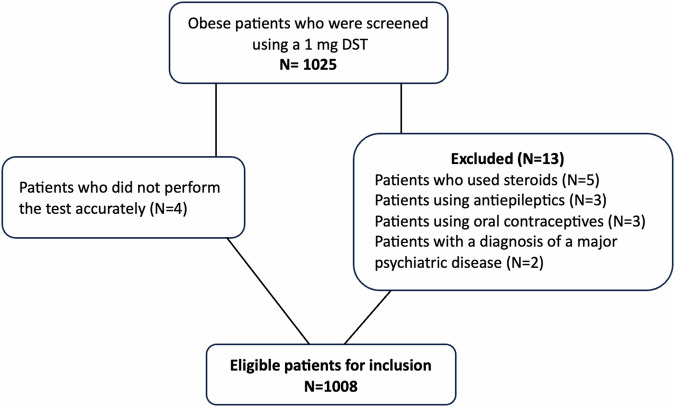
The flow chart of patient inclusion.

This study was conducted as a retrospective study at Diskapi Yildirim Beyazit Training and Research Hospital (newly affiliated name Ankara Etlik City Hospital) Endocrinology Outpatient Clinic. A total of 1025 consecutive patients with obesity (BMI ≥ 30 kg/m²) who were admitted to our outpatient clinic and screened for CS with a 1 mg DST between December 2020 and June 2022 were evaluated for inclusion. World Health Organization classification was used to determine obesity [9]. Exclusion criteria included conditions that are not recommended for performing a 1 mg DST due to potential interference with the results, such as exogenous steroid administration, pregnancy, major psychiatric diseases, and drug use that could affect the clearance of dexamethasone. The patients who did not perform the test accurately were also excluded. After excluding 17 patients with the aforementioned conditions, a total of 1008 eligible patients were included in the study. The flow chart of patient inclusion is presented in Fig. 1.

### Obesity phenotypes

Comorbidities, such as DM, prediabetes, hypertension, dyslipidemia, and coronary artery diseases, were obtained from patient records. The diagnoses were verified by checking the medication use for these diseases and the laboratory parameters of the patients.

MHO was ascertained in patients with obesity whom BMI ≥ 30 kg/m² but did not have any of the following comorbidities: diabetes mellitus (DM), prediabetes, hypertension, coronary artery disease, or dyslipidemia (defined as triglycerides ≥ 150 mg/dL and/or high-density lipoprotein cholesterol (HDL) < 50 mg/dL in women and <40 mg/dL in men) [10]. Other patients, who have any of the comorbidities listed above, are defined as the metabolically unhealthy obesity group (MUO).

Demographic variables, biochemical test results including fasting plasma glucose, HbA1c, triglyceride, total cholesterol, HDL cholesterol, low-density lipoprotein cholesterol (LDL), alanine aminotransferase, creatinine, and thyroid-stimulating hormone levels of all the patients were recorded.

### Screening and diagnostic evaluation

The 1 mg DST was conducted by administering one milligram of dexamethasone orally at 11:00 and obtaining a fasting blood sample for cortisol level measurement between 08:00 and 09:00 AM on the following day. Cortisol levels are measured using the electrochemiluminescence immunoassay method, with the minimum detectable cortisol level being 0.109 mcg/dL. A serum cortisol level of ≤ 1.8 mcg/dL was considered indicative of suppression. Additional confirmation tests, including adrenocorticotropic hormone, midnight serum and salivary cortisol, 24-h urinary cortisol, 2-day 2 mg DST, and imaging results, were also documented based on hospital records for patients who did not achieve cortisol suppression. Patients who do not exhibit any specific clinical signs of Cushing’s syndrome and have cortisol levels ≥ 1.8 mg/dL after the 1 mg dexamethasone suppression test (DST), but hypercortisolism is not verified with the other confirmation tests mentioned above, are defined as having subclinical hypercortisolism [11, 12].

### Statistical analysis

The variables were assessed through both visual methods (such as histograms and probability plots) and analytical methods (specifically, the Kolmogorov-Smirnov and Shapiro-Wilk tests) to determine their normal distribution. To compare non-normally distributed variables, we employed the Mann-Whitney U test, while normally distributed variables were compared using independent sample T-tests. Categorical variables were compared using the Chi-square test or Fisher’s exact test when Chi-square assumptions were not met due to low expected cell counts. The categorical variables were presented using numbers and percentages, normally-distributed variables as means ± standard deviations, and non-normally distributed variables as medians and minimum-maximum values. A *p*-value less than 0.05 was considered statistically significant. Sensitivity is calculated by dividing the number of true positive CS cases by the sum of true positive and false negative cases. Specificity is determined by dividing the number of true negative CS cases by the total of true negative and false positive test results. A significance level of 5% for type-I errors was applied to determine statistical significance.

## Results

The mean age of the patients was 40 ± 12.3 years, and 835 (82.8%) of all the patients were female. The median BMI in the whole study cohort was 43.7 (30.2–65.7) kg/m². Out of 1008 patients, 779 (77.3%) were in the MUO group. Within this group, 180 (23.1%) patients had DM, 225 (28.9%) had prediabetes, 193 (24.8%) had hypertension, 33 (4.2%) had coronary artery disease, and 611 (78.4%) had dyslipidemia. Age, BMI, fasting plasma glucose, HbA1c, triglyceride, and LDL cholesterol levels were found to be higher, while HDL cholesterol levels were lower in the MUO group compared to the MHO group, as expected. Cortisol levels following the 1 mg DST were also significantly higher in the MUO group than in the MHO group (*p* = 0.001). The comparison of demographical data and laboratory test results between the MHO and MUO groups is presented in Table 1.

**Table 1:** The comparison of demographics and laboratory test results between metabolically healthy and unhealthy patients with obesity.

	Patients with MHO (*N* = 229)	Patients with MUO (*N* = 779)	*P*-value
Age, years	35.4 ± 11.1	41.2 ± 12.4	**<0.001**
Female gender, n (%)	202 (87.4)	633 (81.5)	**0.037**
BMI, kg/m^2^	41.4 (31–59.5)	43.5 (30–65.7)	**<0.001**
FPG, mg/dl	87 (65–100)	96 (65–391)	**<0.001**
HbA1c, %	5.5 (4–5.7)	5.9 (4–15)	**<0.001**
Creatinine, mg/dl	0.7 (0.4–1.2)	0.75 (0.39–1.4)	**0.010**
ALT, IU/L	19 (7–89)	22 (6–270)	**<0.001**
Total cholesterol, mg/dl	188 (96–353)	190 (94–424)	0.074
Trygliceride, mg/dl	106 (44–149)	162 (48–748)	**<0.001**
LDL, mg/dl	116 (39–249)	132 (38–262)	**<0.001**
HDL, mg/dl	56 (46–93)	43 (22–98)	**<0.001**
TSH, mIU/L	2.19 (0.1–27)	2.12 (0.1–15)	0.699
1 mg DST cortisol, mcg/dl	0.54 (0.12–5.06)	0.59 (0.16–13.9)	**0.001**

*ALT* Alanine aminotransferase, *BMI* Body mass index, *DST* Dexamethasone suppression test, *FPG* Fasting plasma glucose, *HDL* High-density lipoprotein cholesterol, *LDL* Low-density lipoprotein cholesterol, *MHO* Metabolically healthy obesity, *MUO* Metabolically unhealthy obesity, *TSH* Thyroid-stimulating hormone.

The bold values show significant results.

Among the entire study cohort, 12 (1.2%) patients had a cortisol level ≥ 1.8 following a 1 mg DST. Out of these 12 patients, 11 were in the MUO group. Dyslipidemia, followed by abnormal glucose metabolism disorders and hypertension, were the most common comorbidities observed in patients with unsuppressed cortisol levels after the 1 mg DST. Seven out of the 12 patients had at least two different comorbidities in the MUO phenotype. Within all the patients hypercortisolism was definitively diagnosed in two patients, representing a prevalence of 0.2%. One of these patients had unilateral adrenal lesion, while the other had pituitary microadenoma, which were identified during further diagnostic evaluation, leading to CS. The remaining 10 patients, who had unsuppressed cortisol levels after the 1 mg DST and did not demonstrate any clinical signs of Cushing’s syndrome, were classified as the ones having subclinical hypercortisolism. Within them one patient exhibited unsuppressed cortisol levels following both a 1 mg and a 2-day 2 mg DST. However, salivary and 24-h urine cortisol levels were found to be within normal ranges. This patient continues to be monitored for subclinical CS with no visualized lesions in either the pituitary or adrenal glands. Cortisol levels after 2-day 2 mg DST of other patients with subclinical hypercortisolism have been detected under 1.8 mg/dL, and they continue to be followed up in our clinic as well. The details of further diagnostic tests for patients who had unsuppressed cortisol levels following the 1 mg DST are shown in Table 2. The 1 mg DST exhibited a specificity of 99% and a sensitivity of 100% for the screening of CS in our study cohort.

**Table 2:** The data of the patients with unsuppressed cortisol levels following the 1 mg DST.

	P1	P2	P3	P4	P5	P6	P7	P8	P9	P10	P11	P12
Age	48	43	32	50	42	51	23	43	37	42	18	40
Gender	M	M	M	F	F	F	F	F	M	F	F	F
DM/Prediabetes	+	–	+	+	+	–	–	+	–	+	+	–
Hypertension	–	–	–	+	–	–	+	+	–	+	–	–
Dyslipidemia	+	+	+	–	+	+	+	+	+	+	–	–
CAD	–	–	–	–	–	–	–	–	–	–	–	–
1-mg DST cortisol, mcg/dl	2.98	3	2.35	3.41	**7.1**	4.39	2.49	**13.9**	**5.06**	2.2	4.1	3.1
2 days 2-mg DST cortisol, mcg/dl	0.53	1.45	0.65	1.31	**3.65**	0.67	0.21	**10.8**	**2.07**	0.61	1.22	1.76
ACTH, ng/L	–	21	–	10.5	**90.5**	–	–	**1.8**	14	–	19	12.1
Late night salivary cortisol, ng/ml	–	–	–	–	**2.74**	–	–	**3.1**	1.2	–	–	–
24-h urine cortisol, mcg/24 st	–	–	–	–	**82.5**	15.1	–	33.5	8.5	–	–	11.8
Midnight cortisol, mcg/dl	–	–	–	–	**5.2**	–	–	**9.6**	–	–	–	–
Etiology					Pituitary adenoma			Adrenal adenoma	No lesion			

*ACTH* Adrenocorticotropic hormone, *CAD* Coronary artery disease, *DM* Diabetes mellitus, *DST* Dexamethasone suppression test, *F* Female, *M* Male.

Normal ranges: Salivary cortisol: 0.7–2.2 ng/mL, 24-h urinary cortisol: 3.5–45 mcg/24 h.

## Discussion

The present study demonstrated higher serum cortisol levels after 1 mg DST in the MUO group compared to the MHO group. Within the entire study population, 1.2% of the patients had unsuppressed cortisol levels after the 1 mg DST, with all except one exhibiting a MUO phenotype. The overall prevalence of CS was found to be 0.2% in the whole cohort.

Obesity, as an important clinical feature of CS, shares many overlapping features with this rare endocrinological disease [13]. Consequently, despite the ongoing discrepancies in studies evaluating the necessity of CS screening in the population with obesity, many clinicians continue to perform routine screening using the methods recommended in current guidelines.

A wide range of CS prevalence has been reported in the literature for various populations with obesity. A study involving 369 patients with obesity, which screened for CS using two or three tests including the 1 mg DST, 24-h urine cortisol, and late-night salivary cortisol, did not diagnose any cases of CS [14]. Another study, which included 387 patients with obesity screened with a 1 mg DST and 182 with two separate 24-h urine cortisol tests, also found no patients with CS [15]. The low prevalence of CS was found to be 0.6 and 0.8% in two different studies involving 433 and 783 consecutive morbidly patients with obesity who underwent bariatric surgery [16, 17]. In another extensive cohort study, which included 1037 patients with class 3 obesity screened with a 1 mg DST before bariatric surgery, a 0.77% prevalence of CS was reported, demonstrating a high specificity for DST of 96.8% [18]. In contrast to these low rates, a study from Turkey reported a significant CS rate of 9.33% among individuals with obesity [19]. A more recent study also revealed a higher CS rate of 5.4% in an evaluation of 813 patients with obesity [8]. Our study found a very low CS rate of 0.2% among patients with obesity. Based on all these studies, the general consensus is against screening for CS in individuals with simple obesity alone. Our results also support this recommendation. The specificity of the DST in screening for CS among individuals with obesity is indeed very high, and our results corroborate this observation with a specificity of 99% and a sensitivity of 100% [18]. In our view, employing the 1 mg DST as a screening tool in appropriate patients, as suggested in recent guidelines, has resulted in a slightly higher sensitivity and specificity than other studies. The results of the present study align with previous research, confirming that the 1 mg DST is an appropriate test for CS screening in patients with obesity, demonstrating high sensitivity and specificity rates.

However, obesity is one of the major characteristics of CS, and many clinical findings of obesity and CS overlap. Therefore, it is essential to determine which of these clinical findings and comorbidities are worth screening when they coexist with obesity. The relationship between CS and certain other clinical conditions associated with obesity, such as DM, hypertension, and osteoporosis, has also been previously questioned. The prevalence CS in the diabetic population has been reported to be around 3% in earlier studies, although some of these studies did not identify any cases of CS in patients with DM [20, 21]. However, this rate increased to 9.4% when hospitalized diabetic patients were analyzed [22]. Another study, including a large cohort of 817 patients with type 2 DM, revealed a relatively lower rate of CS at 0.7% when screened using a 1 mg DST [23]. CS rate was found to be 0.5% in a large cohort of 4429 subjects with hypertension using a 1 mg DST as the screening method [24]. In patients with resistant hypertension, 8% have been diagnosed with subclinical hypercortisolism, while among hypertensive patients under the age of 40, 7.5% have been diagnosed with CS [25, 26] A recent study investigating who should be screened for CS demonstrated that while obesity is a key clinical feature, recent weight gain is even more prominent in CS [27]. This study also revealed that osteoporosis and metabolic syndrome are more frequent in patients with CS compared to those in whom CS is ruled out. The study that revealed a 5.4% CS prevalence in obesity suggested that a more reasonable approach for screening should target patients with a BMI between 30 and 34.9 kg/m², aged older than 50 years, and with uncontrolled DM and hypertension [8]. Osteoporosis is seen in nearly half of the patients diagnosed with CS [13]. Nearly a 5% prevalence of subclinical hypercortisolism was reported in patients with osteoporosis who did not exhibit clinical features of CS or secondary osteoporosis [28, 29]. The prevalence of CS appears to be higher in advanced chronic diseases. Nevertheless, having unique comorbidities without the clinical features of CS or being diagnosed at the expected age may not be sufficient for screening, similar to obesity alone.

MHO is a recent nomenclature defining individuals with a BMI higher than 30 kg/m² but who are free from obesity-related comorbidities such as glucose metabolism impairments, hypertension, dyslipidemia, and coronary artery diseases [10]. These patients are characterized by lower visceral and liver fat but higher subcutaneous leg fat content [10]. In addition to this distinct fat distribution compared to MUO, a healthy adipose tissue function, balanced adipokine secretion, and lower inflammatory mediators contribute to their healthy metabolic status [30]. However, it’s important to note that despite being classified as MHO at any age, it is generally considered a transient state of obesity. Studies have shown that metabolic impairments can develop in MHO individuals over a decade of follow-up [31, 32]. To the best of our knowledge, CS screening has not been investigated based on metabolic health status before. According to our results, most of the patients with unsuppressed cortisol levels and those diagnosed with CS were in the MUO group. Dyslipidemia, followed by abnormal glucose metabolism disorders and hypertension, were the most common comorbidities observed in patients with unsuppressed cortisol levels after the 1 mg DST. Furthermore, nearly 60% of these patients had two or more different comorbidities in the MUO phenotype. Only one patient presented MHO phenotype within the patients with subclinical hypercortisolism. A general evaluation under the term of MHO may be a more practical approach than considering separate diseases in obesity when deciding which patients with obesity should be screened for CS.

This study has limitations, including its single-center design and the inclusion of patients from a single racial group. Furthermore, our results could be enhanced by analyzing the presence of clinical features suggestive of CS in patients and by obtaining information on fat distribution. The retrospective design prevented us from retrieving all of these findings. However, the strict exclusion criteria, the focus on analyzing patients suggested screening with a 1 mg DST according to current guidelines, and the inclusion of a considerable number of patients are strengths of this study.

In conclusion, while the 1 mg DST proves to be a practical screening tool for CS with high sensitivity and specificity in individuals with obesity, the number of CS cases detected remains relatively low. Screening all patients with obesity for CS without considering any associated metabolic conditions appears impractical and unnecessary in everyday clinical practice. However, it may be more reasonable and applicable to selectively screen the patients with obesity having comorbidities such as DM, hypertension, dyslipidemia, or coronary artery disease, which lead to a metabolically unhealthy phenotype, rather than all individuals with obesity.

## Data Availability

The datasets generated during and/or analysed during the current study are available from the corresponding author on reasonable request.

## References

[1] Reincke M, Fleseriu M. Cushing syndrome. JAMA. 2023;330:170. 37432427 10.1001/jama.2023.11305

[2] Rubinstein G, Osswald A, Hoster E, Losa M, Elenkova A, Zacharieva S, et al. Time to diagnosis in Cushing’s syndrome: a meta-analysis based on 5367 patients. J Clin Endocrinol Metab. 2020;105:e12–22. 10.1210/clinem/dgz136 31665382

[3] Nieman LK, Biller BMK, Findling JW, Newell-Price J, Savage MO, Stewart PM, et al. The diagnosis of Cushing’s syndrome: an endocrine society clinical practice guideline. J Clin Endocrinol Metab. 2008;93:1526–40. 18334580 10.1210/jc.2008-0125 PMC2386281

[4] Sims EAH. Are there persons who are obese, but metabolically healthy? Metabolism. 2001;50:1499–504. 11735101 10.1053/meta.2001.27213

[5] Stefan N. Identification and characterization of metabolically benign obesity in humans. Arch Intern Med. 2008;168:1609. 18695074 10.1001/archinte.168.15.1609

[6] Stefan N, Häring H-U, Schulze MB. Metabolically healthy obesity: the low-hanging fruit in obesity treatment? Lancet Diabetes Endocrinol. 2018;6:249–58. 28919065 10.1016/S2213-8587(17)30292-9

[7] Lammert A, Nittka S, Otto M, Schneider-Lindner V, Kemmer A, Krämer BK, et al. Performance of the 1 mg dexamethasone suppression test in patients with severe obesity. Obesity. 2016;24:850–5. 26948683 10.1002/oby.21442

[8] Atar RV. The frequency of Cushing’s disease, ACTH independent Cushing’s syndrome, and autonomous cortisol secretion among Turkish patients with obesity. North Clin Istanb. 2020;7:214–21. 32478291 10.14744/nci.2019.54771 PMC7251263

[9] Obesity: preventing and managing the global epidemic. Report of a WHO consultation. World Health Organ Tech Rep Ser. 2000;894:i–xii, 1–253. 11234459

[10] Blüher M. Metabolically healthy obesity. Endocr Rev. 2020;41:bnaa004. 32128581 10.1210/endrev/bnaa004 PMC7098708

[11] Chiodini I. Diagnosis and treatment of subclinical hypercortisolism. J Clin Endocrinol Metab. 2011;96:1223–36. 21367932 10.1210/jc.2010-2722

[12] Prete A, Bancos I. Mild autonomous cortisol secretion: pathophysiology, comorbidities and management approaches. Nat Rev Endocrinol. 2024;20:460–73. 38649778 10.1038/s41574-024-00984-y

[13] Shimon I. Screening for Cushing’s syndrome: Is it worthwhile? Pituitary. 2015;18:201–5. 25578150 10.1007/s11102-015-0634-9

[14] Baid SK, Rubino D, Sinaii N, Ramsey S, Frank A, Nieman LK. Specificity of screening tests for Cushing’s syndrome in an overweight and obese population. J Clin Endocrinol Metab. 2009;94:3857–64. 19602562 10.1210/jc.2008-2766 PMC2758724

[15] Glyn TC, Ho MW, Lambert AP, Thomas JDJ, Douek IF, Andrews RC, et al. Patients with morbid obesity should not be routinely screened for Cushing’s syndrome: results of retrospective study of patients attending a specialist weight management service. Clin Obes. 2020;10:e12358. 31994330 10.1111/cob.12358

[16] Janković D, Wolf P, Anderwald C-H, Winhofer Y, Promintzer-Schifferl M, Hofer A, et al. Prevalence of endocrine disorders in morbidly obese patients and the effects of bariatric surgery on endocrine and metabolic parameters. Obes Surg. 2012;22:62–9. 22052199 10.1007/s11695-011-0545-4

[17] Fierabracci P, Pinchera A, Martinelli S, Scartabelli G, Salvetti G, Giannetti M, et al. Prevalence of endocrine diseases in morbidly obese patients scheduled for bariatric surgery: beyond diabetes. Obes Surg. 2011;21:54–60. 20953730 10.1007/s11695-010-0297-6

[18] Yavuz DG, Apaydin T, Gunhan HG, Uygur MM. Assessment of 1 mg dexamethasone suppression test in patients with obesity before bariatric surgery. Obes Surg. 2020;30:4981–5. 32803707 10.1007/s11695-020-04865-x

[19] Tiryakioglu O, Ugurlu S, Yalin S, Yirmibescik S, Caglar E, Yetkin DO, et al. Screening for Cushing’s syndrome in obese patients. Clinics. 2010;65:9–13. 20126340 10.1590/S1807-59322010000100003 PMC2815288

[20] Leibowitz G, Tsur A, Chayen SD, Salameh M, Raz I, Cerasi E, et al. Pre-clinical Cushing’s syndrome: an unexpected frequent cause of poor glycaemic control in obese diabetic patients. Clin Endocrinol. 1996;44:717–22. 10.1046/j.1365-2265.1996.737558.x 8759185

[21] Mullan K, Black N, Thiraviaraj A, Bell PM, Burgess C, Hunter SJ, et al. Is there value in routine screening for Cushing’s syndrome in patients with diabetes? J Clin Endocrinol Metab. 2010;95:2262–5. 20237165 10.1210/jc.2009-2453

[22] Chiodini I, Torlontano M, Scillitani A, Arosio M, Bacci S, Di Lembo S, et al. Association of subclinical hypercortisolism with type 2 diabetes mellitus: a case-control study in hospitalized patients. Eur J Endocrinol. 2005;153:837–44. 16322389 10.1530/eje.1.02045

[23] Terzolo M, Reimondo G, Chiodini I, Castello R, Giordano R, Ciccarelli E, et al. Screening of Cushing’s syndrome in outpatients with type 2 diabetes: results of a prospective multicentric study in Italy. J Clin Endocrinol Metab. 2012;97:3467–75. 22767639 10.1210/jc.2012-1323

[24] Anderson GH, Blakeman N, Streeten DHP. The effect of age on prevalence of secondary forms of hypertension in 4429 consecutively referred patients. J Hypertens. 1994;12:609. 7930562 10.1097/00004872-199405000-00015

[25] Martins LC, Conceição FL, Muxfeldt ES, Salles GF. Prevalence and associated factors of subclinical hypercortisolism in patients with resistant hypertension. J Hypertens. 2012;30:967–73. 22406465 10.1097/HJH.0b013e3283521484

[26] Trifanescu R, Carsote M, Caragheorgheopol A, Hortopan D, Dumitrascu A, Dobrescu M, et al. Screening for secondary endocrine hypertension in young patients. Maedica. 2013;8:108–15. 24371473 PMC3865118

[27] Braun LT, Vogel F, Zopp S, Marchant Seiter T, Rubinstein G, Berr CM, et al. Whom should we screen for Cushing syndrome? The Endocrine Society Practice Guideline Recommendations 2008 Revisited. J Clin Endocrinol Metab. 2022;107:e3723–30. 35730067 10.1210/clinem/dgac379 PMC9387700

[28] Chiodini I, Mascia ML, Muscarella S, Battista C, Minisola S, Arosio M, et al. Subclinical hypercortisolism among outpatients referred for osteoporosis. Ann Intern Med. 2007;147:541. 17938392 10.7326/0003-4819-147-8-200710160-00006

[29] Kann P, Laudes M, Piepkorn B, Heintz A, Beyer J. Suppressed levels of serum cortisol following high-dose oral dexamethasone administration differ between healthy postmenopausal females and patients with established primary vertebral osteoporosis. Clin Rheumatol. 2001;20:25–9. 11254236 10.1007/s100670170099

[30] Stefan N, Häring H-U, Hu FB, Schulze MB. Metabolically healthy obesity: epidemiology, mechanisms, and clinical implications. Lancet Diabetes Endocrinol. 2013;1:152–62. 24622321 10.1016/S2213-8587(13)70062-7

[31] Mongraw-Chaffin M, Foster MC, Anderson CAM, Burke GL, Haq N, Kalyani RR, et al. Metabolically healthy obesity, transition to metabolic syndrome, and cardiovascular risk. J Am Coll Cardiol. 2018;71:1857–65. 29699611 10.1016/j.jacc.2018.02.055 PMC6002856

[32] Lin H, Zhang L, Zheng R, Zheng Y. The prevalence, metabolic risk and effects of lifestyle intervention for metabolically healthy obesity. Medicine. 2017;96:e8838. 29381992 10.1097/MD.0000000000008838 PMC5708991

